# Pull-off bond strength of orthodontic molar bands cemented to enamel with novel calcium silicate, glass ionomer, and zinc phosphate cement

**DOI:** 10.2340/biid.v13.46290

**Published:** 2026-06-24

**Authors:** Mariantonietta Leo, Ishan El Khadery, Michel Dalstra, Dirk Leonhardt, Bahram Ranjkesh, Henrik Løvschall

**Affiliations:** a Department of Clinical Sciences and translational Medicine, Tor Vergata University of Rome, Rome, Italy; b Department of Dentistry and Oral Health, Aarhus University-Health, Aarhus, Denmark

**Keywords:** Calcium silicate cement, pull‑off bond strength, orthodontic bands, in vitro, dental biomaterials

## Abstract

**Aim:**

This in vitro study compared the pull‑off bond strength of orthodontic molar bands cemented to enamel using a novel fast‑setting and bioactive calcium silicate cement (CSC), a glass ionomer cement (GIC), and a zinc phosphate cement after 1 month of storage in phosphate‑buffered saline (PBS).

**Materials and Methods:**

Ninety extracted human molars were randomly allocated to three groups and fitted with orthodontic molar bands. Bands were cemented using a novel fast‑setting CSC (Protooth), a GIC (Orthocem B), or a zinc phosphate cement (DeTrey Zinc) according to manufacturers’ instructions. After storage in PBS for 28 days, band debonding was performed using a universal testing machine in a wire‑loop pull‑off configuration at a cross‑head speed of 1 mm/min. The maximum load at failure was divided by the band surface area to calculate pull‑off bond strength (MPa). Data were analyzed using the Kruskal–Wallis test followed by pairwise Mann–Whitney U tests with Holm–Bonferroni correction (α = 0.05).

**Results:**

The median pull‑off bond strength of the CSC was 1.22 MPa (interquartile range [IQR]: 0.93–1.34 MPa), which was significantly higher than that of the GIC 0.50 MPa (IQR: 0.33–0.79 MPa; *p* < 0.05). No statistically significant difference was observed between the CSC and zinc phosphate cement 0.92 MPa (IQR: 0.61–1.23 MPa; *p* ≥ 0.05). All specimens exhibited similar failure modes, with less than 30 % cement remaining on enamel following debonding.

**Conclusions:**

Within the limitations of this in vitro study, the novel fast‑setting CSC demonstrated pull‑off bond strength similar to zinc phosphate cement and significantly higher than GIC after 1 month of storage in PBS. Further studies are required to evaluate the clinical performance and long‑term behavior of this material.

**KEY MESSAGES**

1. The novel fast‑setting and bioactive calcium silicate cement (Protooth) produced pull-off bond strengths for orthodontic molar bands that were significantly higher than glass ionomer cement and similar to zinc phosphate cement after 1 month in physiological-like liquid (PBS).

## Introduction

New dental materials are being developed to enhance oral health thanks to ongoing technological research. Secure attachment of molar bands to archwires is essential for effective orthodontic treatment. Various luting materials are available for bonding bands to teeth, making it important to identify which adhesives provide the most reliable bond while also minimizing the risk of dental decay [1]. This in vitro study intends to evaluate the pull-off bond strength of a novel bioactive calcium silicate cement (CSC) called Protooth. The composition has been formulated as a fast-setting CSC, designed to enhance strength and facilitate practical applications in dental crowns. It stands out due to its bioactivity and content with fluoride [2], which may help promote de novo apatite formation [3], and potentially reduce the risk of demineralization around cemented orthodontic bands.

Bands are typically placed in the posterior region of the mouth, where they endure the greatest forces from mastication [1]. The retention of these bands is crucial for the success of orthodontic treatment, as they are subjected to significant mechanical forces [4]. The strength of the luting cement—specifically its pull-off and tensile strength properties—plays a vital role in maintaining the retention of these devices under masticatory forces. Over the past decades, zinc phosphate cement has been the gold standard test material for orthodontic bands, and new orthodontic cements have been compared to it [1, 4]. The primary retention of zinc phosphate cement is due to the relatively high compressive strength and mechanical bonding between the orthodontic band and tooth enamel. However, chemical bonding to the enamel is lacking. Other drawbacks of zinc phosphate cement include low tensile strength, brittleness, short working time, and high solubility in the oral cavity, which causes microleakage [1, 4].

Glass ionomer cement (GICs) has been widely used in clinical dentistry for decades [5]. They can be used for lining, bonding, luting, sealing, or restorations. Compared with resin-based composites, the advantages of GICs include fluoride release to support caries control, relatively high biocompatibility, adhesion to enamel without requiring an additional adhesive agent, and ease of use [6]. However, their low fracture toughness and moisture sensitivity are major drawbacks [7].

CSCs release calcium hydroxide during setting, which supports bioactive apatite formation in physiological-like environments and has bacteriostatic effects. Despite this advantage, limitations such as the very long setting time of mixed thin pastes for luting are not optimal for applicability in tooth crowns [8, 9].

Accordingly, it seems interesting to test novel fast-setting CSC with fluoride, which have been developed with mechanical improvements for use in tooth crowns, including cementation. The pull-off bond strength test allows clinicians to measure the resistance of cements to external forces [1]. This study aimed to compare the enamel pull-off bond strength of orthodontic bands cemented with routinely used luting cements, including glass ionomer and zinc phosphate cement, versus a novel fast-setting CSC after 1 month in phosphate-buffered saline (PBS).

## Materials and methods

### Tooth selection and sample preparation

Extracted human molars collected from various dental departments were preserved in a solution of 0.4% thymol. The study confirmed that the teeth were anonymized, with no patient identifiers, ensuring compliance with both institutional and national guidelines. These teeth were sourced from patients who had undergone other dental treatments, in accordance with the Danish National Center for Ethics (Regulation 2017, Version 1.0).

Based on pilot study, a total of 90 extracted upper and lower molars were chosen as in vitro experimental models, excluding teeth with fillings on the proximal or oral surfaces and teeth that were severely destroyed.

The teeth were cleaned with a hand instrument to remove any remaining soft tissue still attached to them. They were allocated using block randomization, with blocks balanced for size and form, and were then randomly assigned within each block to test groups for band cementation using zinc phosphate cement, GIC, or a novel CSC. Subsequently, each tooth was embedded in a plastic cylinder with a cold-setting pink acrylic resin, which covered the tooth from the root apexes to the cemento-enamel junction. It was taken into consideration to keep the tooth parallel to its vertical axis, where all surfaces of the teeth were visible when looking to the tooth from occlusal surface with naked eyes. After setting up the acrylic resin, the embedded tooth specimen was cleaned to remove residual acrylic resin that may have affected the tooth surfaces, and the teeth were polished with pumice.

A metallic cylinder was designed to fit around the plastic cylinder containing the embedded tooth. Two 1.5-mm-diameter holes were created on opposite sides near the top, close to the furcation area. During the mechanical pull-off test, steel pins inserted through these holes secured the plastic cylinder and prevented displacement.

### Orthodontic bands selection

The bands (Ormco AOA, California, USA) were selected according to tooth diameter. The bands with two attachments on both the facial and palatal sides were selected according to the best fit. The band was fitted to the tooth periphery, adapted to the tooth without difficulty, and cementated with minimal finger pressure. The bands were selected and placed by the same operator to eliminate any operator bias in the band positioning and fitting.

### Tested cements

Three types of cements were tested: zinc phosphate cement (DeTrey Zinc, Dentsply, Germany), GIC (Orthocem B, PSP Dental, UK, used for orthodontic band cementation at the orthodontic department at Aarhus University), and a novel fast-setting CSC (Protooth, Dentosolve, Denmark). Orthodontic bands were cemented according to the manufacturer’s instructions. Zinc phosphate cement and GIC were mixed manually on a mixing pad.

The Protooth composition tested in this study was designed with radiocontrast properties, as well as adjusted consistency and fast-setting comparable to those of zinc phosphate cement. The Protooth cement included tricalcium silicate, dicalcium silicate, nano-SiO_2_, tricalcium aluminate, calcium sulfate, fluoride additive (3.5%), and (10%) ZrO_2_ as a radiocontrast. Protooth cement was mixed by placing 1 g of cement in a capsule and adding the mixing liquid using a pipette (208 µL). The capsule was closed, and inserted into an amalgam-mixing machine for 20 s.

**Figure 1:**
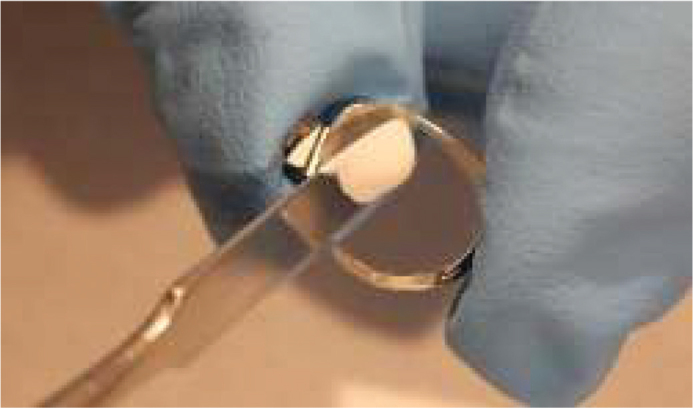
The band was filled with Protooth mixed to a creamy paste consistency.

After mixing, each cement was applied to the orthodontic band with a spatula (Figure 1), and the band was seated on the tooth with finger pressure. Excess cement in the occlusal and cervical areas was removed before the cement was set.

**Figure 2:**
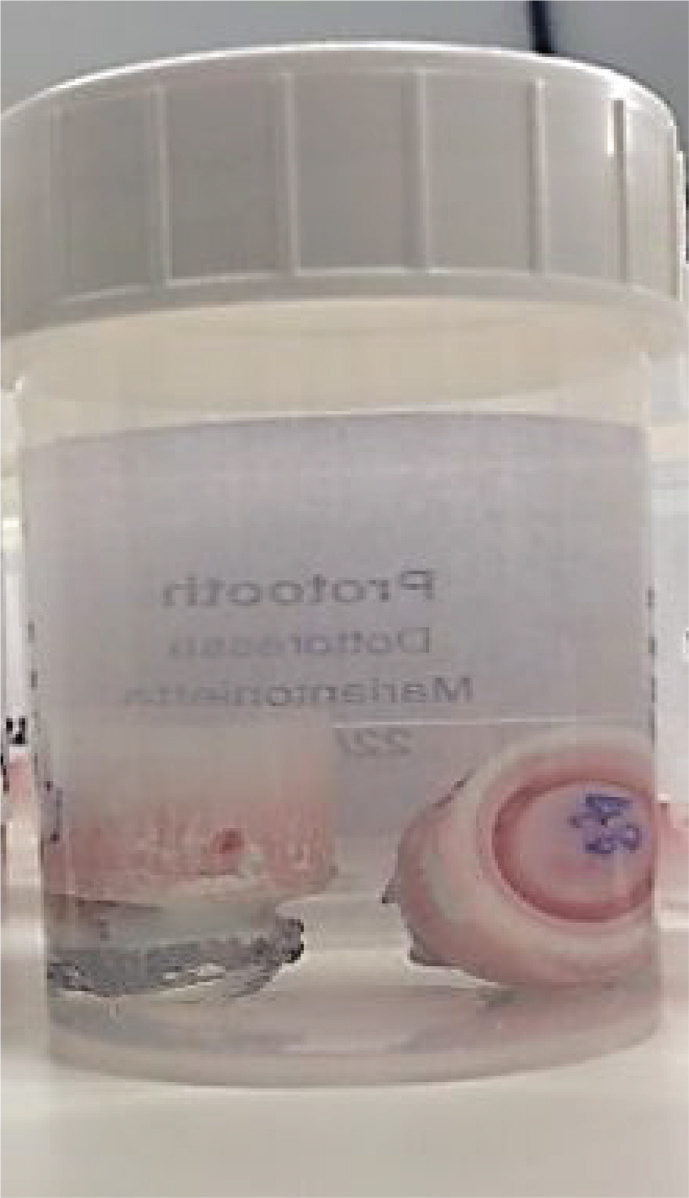
After setting of the cements, all teeth were kept in a humid environment in an incubator at 37°C for 48 h. The teeth were then transferred to PBS in a container (in the figure) and kept in this solution for 28 days to simulate a physiologically similar environment that can occur naturally in the oral cavity by saliva.

All teeth were kept in a humid environment in an incubator at 37°C for 48 h (Figure 2). The teeth were then transferred to PBS and kept in this solution for 28 days to simulate a physiologically similar environment that can occur naturally in the oral cavity by saliva. PBS contained 136.4 mmol/l NaCl, 2.7 mmol/L KCL, 8.2 mmol/l NaH_2_PO_4_, and 1.25 mmol/L KH_2_PO_4_ in 1,000 mL of distilled water at PH 7.4.

### Pull-off bond strength testing

**Figure 3:**
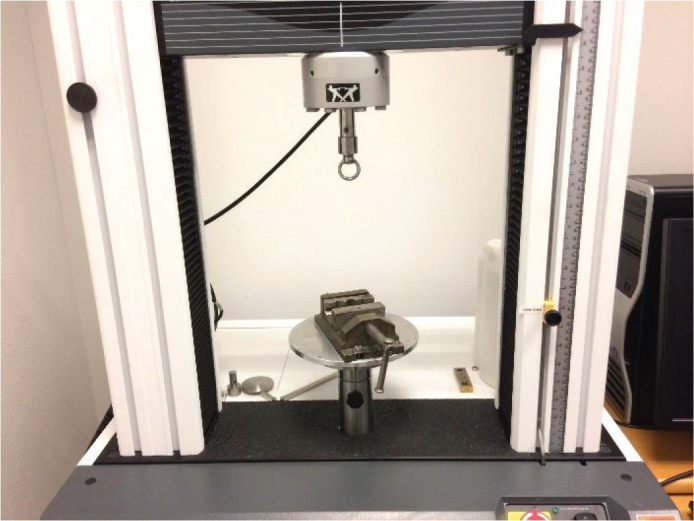
Universal Testing Machine (Instron, Canton, MA, USA)

**Figure 4:**
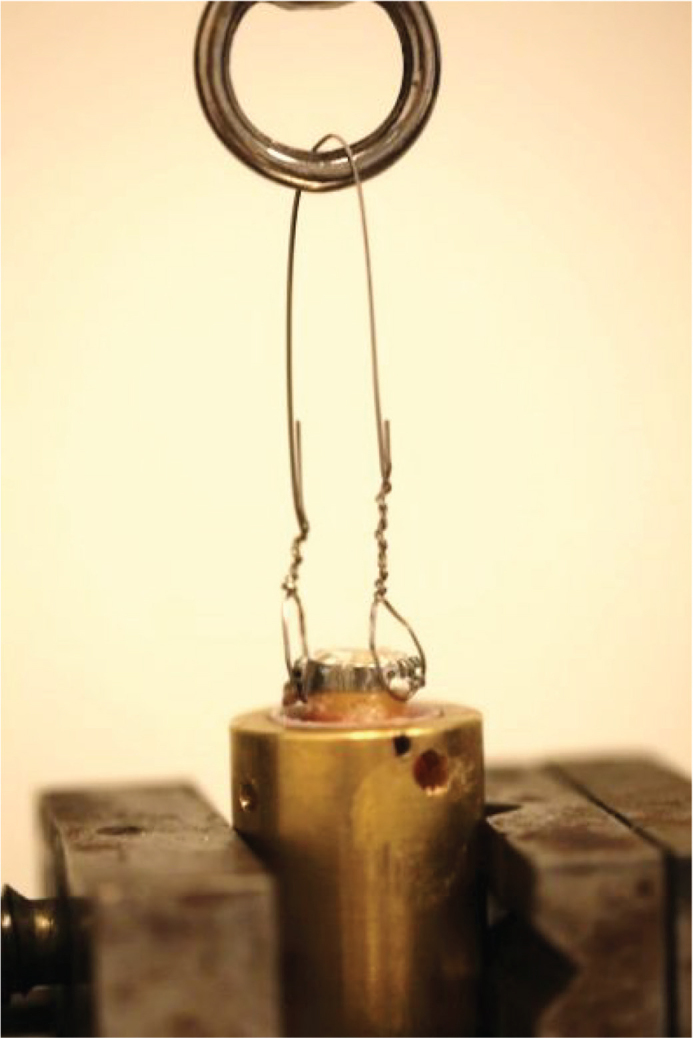
The loose wire through the ring makes it possible to balance the pulling force on both sides.

Pull-off bond strength was measured with a Universal Testing Machine (Instron, Canton, MA, USA) (Figure 3) [10] using a stainless steel wire (0.016 *0.022 in.) (Figure 4). A pull-off force with cross-head speed of 1 mm/min was applied to the band until failure of the cementation, and the band was detached from the tooth. The same operator performed all debonding. The Bluehill 3 program was used to draw the curve with the force measured in Newtons on the Y-axis and the distance (on the S axis of the diagram). The force in Newtons was correlated with the inner band surface. The wire is pulled loosely through the ring making it possible to balance the pull on both sides of the ring (Figure 4).

As data violated assumptions of normality, overall group differences were assessed using the Kruskal–Wallis test. When significant, post hoc pairwise comparisons were performed using Mann–Whitney U tests. Holm–Bonferroni correction was applied to adjust for multiple comparisons. Unadjusted *p*-values are reported, and significance was determined using sequentially adjusted α-levels. Data were analyzed using IBM SPSS Statistics (IBM Corporation, Armonk, NY, USA).

After testing mechanical cement properties, the type of failure, which categorizes the amount of cement remaining on the tooth surface after band removal, was visually estimated following standardized scoring. Type I: most cement remaining on enamel. Type II: ± 50% remaining on enamel. Type III: less than 30% on enamel according to study by Maijer et al. [11].

## Results

**Figure 5:**
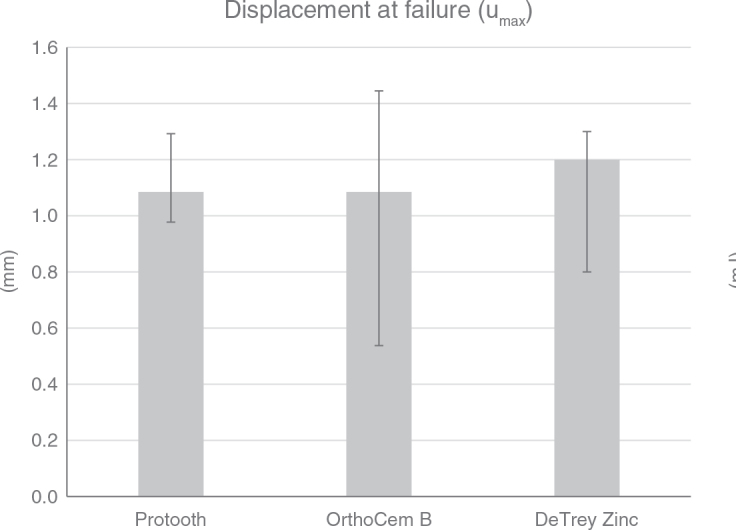
The median and 25% quartiles of the displacement at failure for the three cements.

**Figure 6:**
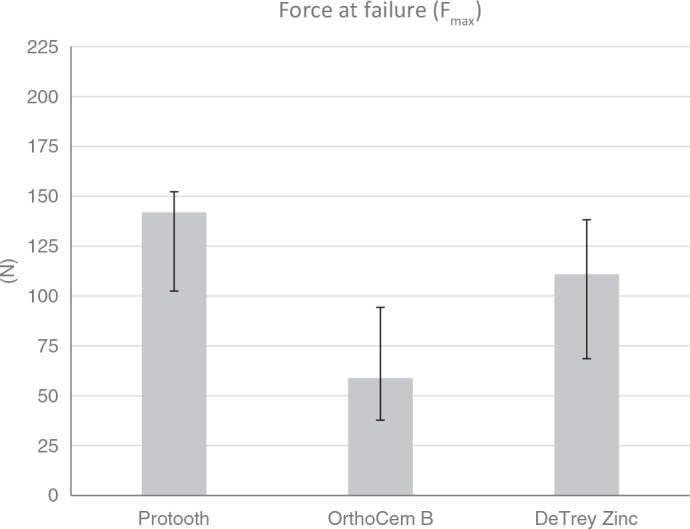
The median and 25% quartiles of force at failure for the three cements.

**Figure 7:**
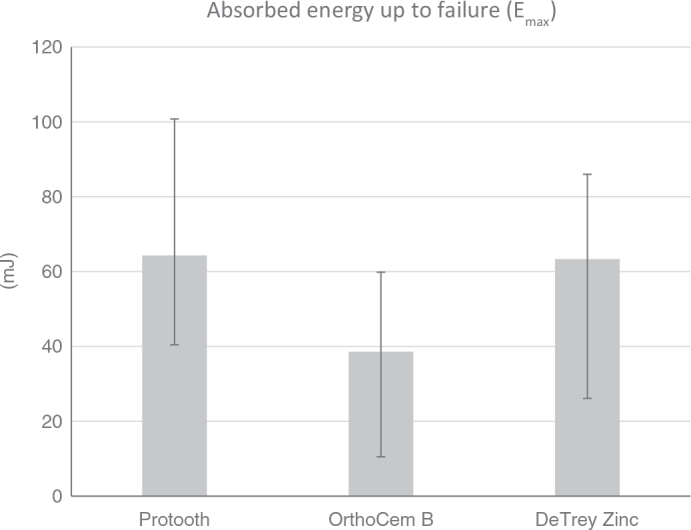
The median and 25% quartiles of absorbed energy up to failure for the three cements.

**Figure 8:**
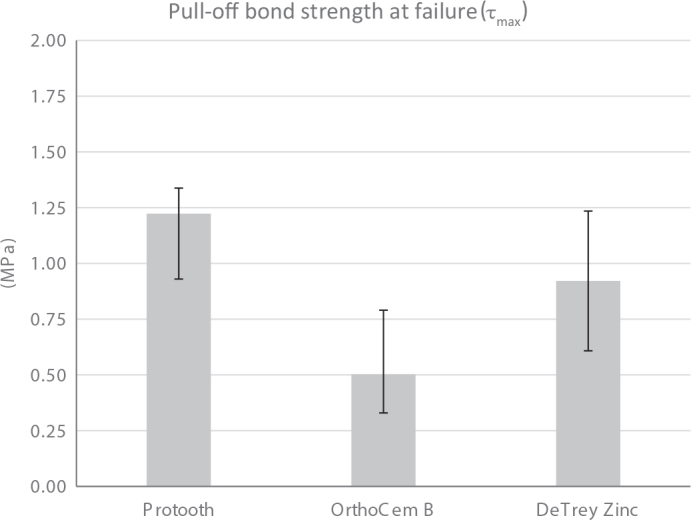
The median and 25% quartiles of the pull-off bond strength (MPa) for the three cements.

The descriptive statistics of the measured pull-off bond strength values are presented in Table 1. Significant group differences were observed for force at failure (p=0.0015, Fmax in Fig. 6) and pull-off bond strength at failure (p = 0.0123, τmax in Fig. 8), but not for displacement at failure (p = 0.620, umax in Fig. 5) or absorbed energy up to failure (p = 0.0641, Emax in Fig. 7). Post hoc Mann–Whitney U tests with Holm–Bonferroni correction revealed significant differences for Fmax and τmax between Protooth versus Orthocem B and Orthocem B versus DeTrey Zinc (*p* < adjusted α), whereas Protooth versus DeTrey Zinc was not significant. No significant pairwise differences were found for umax or Emax.

**Table 1:** Pull-off bond strength of orthodontic band cementation after storage in PBS: Comparison of Orthocem B (OC), DeTrey Zinc (ZP), and Protooth (PT). Median, quartiles, and P-value of the different groups.

	u_max_			F_max_			E_max_			_τmax_		
	Displacement at failure			Force at failure			Absorbed energy up to failure			Pull-off bond strength at failure		
	mm			*N*			mJ			MPa		
	median	1. quartile	3. quartile	median	1. quartile	3. quartile	median	1. quartile	3. quartile	median	1. quartile	3. quartile
Protooth	1.09	0.98	1.29	141.94	102.45	152.26	64.31	40.43	100.78	1.22	0.93	1.34
Orthocem B	1.09	0.54	1.45	58.87	37.77	94.29	38.60	10.52	59.84	0.50	0.33	0.79
DeTrey Zinc	1.20	0.80	1.30	110.95	68.55	138.23	63.35	26.13	86.00	0.92	0.61	1.23
	*p*-value			*p*-value			*p*-value			*p*-value		
PT vs. OC	0.6198			0.0015*			0.0641			0.0123*		
PT vs. ZP	0.9802			0.1203			0.426			0.3279		
OC vs. ZP	0.6304			0.0151*			0.163			0.0143*		

Umax: displacement of the fracture; Fmax: force on the fracture; Emax: mechanical energy of fracture; τmax: pull-off stress as force per band area of fracture.

* Indicates statistical significance after Holm–Bonferroni correction. Non‑significant differences are indicated as *p*-value ≥ 0.05.

After band removal no difference in failure type was observed between the groups. Less than 30% remaining cement on enamel was detected in all samples.

## Discussion

In this in vitro study, the pull-off bond strength of orthodontic molar bands cemented to enamel was evaluated after 1 month of storage in PBS. The main finding was that the novel fast-setting CSC (Protooth) exhibited significantly higher pull-off bond strength than the GIC (Orthocem B). No statistically significant difference was observed between the novel CSC (Protooth) and zinc phosphate cement (DeTrey), indicating comparable mechanical performance under the experimental conditions.

Secure attachment of orthodontic molar bands is essential for successful orthodontic treatment, as posterior bands are exposed to substantial mechanical forces during mastication and appliance activation [1]. Inadequate retention may result in band loosening, formation of micro‑gaps at the enamel–cement interface, and increased plaque accumulation, thereby increasing the risk of enamel demineralization and caries development [12]. Consequently, the evaluation of debonding resistance of orthodontic luting cements remains of clinical relevance.

The experimental setup used in the present study employed a wire‑loop pull‑off configuration, which introduces predominantly tensile and peel stress components at the band–cement–enamel interface rather than pure pull-off stresses. For this reason, the outcome variable was defined as pull‑off bond strength. Although this loading configuration does not reproduce the full complexity of intraoral forces, it allows standardized comparison of debonding resistance across materials under controlled conditions and has relevance for assessing band retention mechanisms [1, 4].

The comparable pull‑off bond strength observed for the CSC and zinc phosphate cement is consistent with previous investigations of fast‑setting CSCs in other dental applications. Earlier studies have reported retentive strength, diametral tensile strength, dentin bond strength, and post-cementation performance comparable to conventional zinc phosphate and GICs [10, 11, 14, 15]. These findings suggest that modifications in CSC composition, including altered particle size distribution, optimized liquid‑to‑cement ratio, and incorporation of specific additives including fluorides, can improve mechanical properties and handling characteristics to levels suitable for luting applications.

In contrast, the GIC exhibited significantly lower pull‑off bond strength compared with both the calcium silicate and zinc phosphate cements. This finding may be influenced by the known moisture sensitivity and lower fracture toughness of conventional GICs, particularly during early maturation [7, 20, 21]. Although GICs provide clinically relevant benefits such as fluoride release and chemical adhesion to enamel [5, 6], their mechanical properties may be compromised under prolonged exposure to aqueous environments, such as the PBS storage used in the present study [23, 24].

Failure mode analysis demonstrated Type III failures (<30 % cement remaining on enamel) in all specimens across all groups. As no variability in failure type was observed, statistical comparison was not applicable. This uniform failure pattern indicates that debonding predominantly occurred at the cement–enamel or cement–band interface and suggests that differences in pull‑off bond strength among the materials were not associated with differences in failure mode [11].

CSCs are known to release calcium hydroxide during hydration and exhibit bioactive properties, including apatite formation when exposed to phosphate‑containing physiological‑like solutions [2, 3, 25]. In previous studies, fluoride‑containing CSCs demonstrated the ability to induce calcium phosphate precipitation and apatite formation over time in PBS [2, 3]. However, it is important to emphasize that the present study did not directly assess bioactivity, ion release, or mineralization at the enamel–cement interface. Therefore, any potential effects related to remineralization or caries prevention cannot be concluded from the current findings and should be addressed in future targeted investigations.

Several limitations of this study must be acknowledged. The in vitro design does not replicate the complex biological, mechanical, and thermal challenges present in the oral environment. Additionally, the pull‑off test configuration does not fully represent the multidirectional forces acting on orthodontic bands in vivo. The storage period was limited to 1 month, and longer‑term aging, thermocycling, or mechanical fatigue may influence the relative performance of the tested cements [23, 24, 26]. Although previous studies have reported comparable findings regarding orthodontic band retention and cement performance [13, 16–19, 22, 27–29], extrapolation of the results in this study to long-term clinical performance should be made with caution.

Within the limitations of this in vitro study, the findings indicate that the novel fast‑setting CSC provides pull‑off bond strength comparable to zinc phosphate cement and higher than GIC after 1 month of storage in PBS. Further laboratory studies and clinical investigations are required to evaluate long‑term performance, aging behavior, and possible clinical implications of this material for orthodontic band cementation.

## Data Availability

The datasets used and/or analyzed during the current study are available from the corresponding author on reasonable request.
